# Multidrug resistance of bacterial pathogens in canine pyometra

**DOI:** 10.1111/jsap.70110

**Published:** 2026-03-16

**Authors:** M. G. M. Camozzi, S. B. Waller, E. C. Castelhano, A. L. S. Neves, B. R. Arrais, J. F. N. Pinto, B. G. Alves, C. N. Moreira

**Affiliations:** ^1^ Programa de Pós‐Graduação em Biociência e Saúde Única (PPGBSU), Instituto de Ciências Agrárias (ICA) Universidade Federal de Jataí (UFJ) Jataí Brazil; ^2^ Núcleo de Biotecnologia Centro de Desenvolvimento Tecnológico (CDTec), Universidade Federal de Pelotas (UFPel) Pelotas Brazil; ^3^ Instituto de Ciências Biológicas Universidade Federal de Jataí UFJ Jataí Brazil; ^4^ Instituto de Ciências da Saúde Universidade Federal de Jataí UFJ Jataí Brazil; ^5^ Conception Biosciences Inc. Berkeley California USA

## Abstract

**Objectives:**

To characterise multidrug antimicrobial resistance and pathogenicity profiles of bacteria isolated from different anatomical sites in bitches with pyometra, emphasising their clinical relevance for diagnosis and therapeutic decision‐making in small animal practice.

**Materials and Methods:**

Bacteria were obtained from the faeces, urine, uterus and vagina of 23 dogs diagnosed with pyometra undergoing ovariohysterectomy. Antimicrobial resistance profiles were analysed for all identified isolates, together with phylogenetic classification of *Escherichia coli* and detection of virulence factor–encoding genes.

**Results:**

Bacterial isolates obtained from faecal, urinary, uterine and vaginal samples showed predominantly positive growth for *Escherichia coli*, followed by *Staphylococcus* spp., *Klebsiella pneumoniae* and *Proteus* spp. Among these isolates recovered from each anatomical site, multidrug‐resistant bacteria – defined as resistance to three or more classes of antimicrobials – were identified across all bacterial genera, with frequencies of 37.7% among faecal isolates, 16.7% among urinary isolates, 44.5% among uterine isolates and 100% among vaginal isolates, except for *Enterobacter* spp. Among dogs positive for *Escherichia coli* (11/23), phylogroups A/C (30.9%) were the most prevalent, followed by D/E (16.9%), B2 (15.5%), B1 (12.7%), A (12.7%), C (8.5%) and U (2.8%). In 90% (10/11) of cases, *Escherichia coli* isolates recovered from faecal, vaginal and/or uterine samples belonged to the same clonal family, suggesting faecal contamination. Overall, 77.4% of *Escherichia coli* isolates of uterine origin were classified as resistant or multidrug‐resistant, and 83.9% harboured one or more virulence factor‐encoding genes (hlyA, uspA, papC and predominantly fimH). Phylogroup B2 was the only group in which all assessed virulence factors were simultaneously detected.

**Clinical Significance:**

The presence of multidrug‐resistant bacteria across all genera isolated in cases of canine pyometra underscores the clinical importance of antimicrobial susceptibility testing. Furthermore, the recovery of genetically related *Escherichia coli* isolates from intestinal and reproductive tract samples supports the intestinal microbiota as a relevant source of infection in affected bitches.

## INTRODUCTION

Pyometra is a systemic disease that causes purulent accumulation in the uterine lumen (Liao et al., 2020). Its incidence in sexually mature female dogs is often associated with hormonal, genetic and bacterial factors (Hagman, 2022) and can present with or without vaginal discharge, fever, depression and anorexia (Lansubsakul et al., 2022). This disease can be fatal due to systemic complications (Liao et al., 2020). While *Escherichia coli* is the most common pathogen (Agostinho et al., 2014), others such as *Staphylococcus* spp., *Pseudomonas* spp. and *Klebsiella* spp. may also be involved (Huber et al., 2022; Lansubsakul et al., 2022). Certain virulence factors (VFs), including adhesins, toxins and iron acquisition systems (Maluta et al., 2014; Mateus et al., 2013), enable commensal uterine bacteria to become pathogenic. Additionally, bacteria originating from faeces, vagina and bladder (Mateus et al., 2013) can ascend and contaminate the uterus due to cervical relaxation caused by oestrogenic action (Tamada et al., 2012).

Pyometra development is closely linked to hormonal changes, particularly during the luteal phase, when increased progesterone levels lead to cystic endometrial hyperplasia and excessive glandular secretion, creating a favourable environment for bacterial proliferation (Hagman, 2022). *Escherichia coli* strains involved in pyometra belong to the extraintestinal pathogenic *E. coli* (ExPEC) group, which has the ability to colonise the intestine as commensals but can also disseminate and infect other sites, including the urinary tract and the uterus (Rubio et al., 2014). These bacteria are highly adapted due to mobile genetic elements, such as plasmids, integrative and conjugating elements, which contribute to their pathogenicity (Denamur et al., 2021).

Although ovariohysterectomy (OSH), antimicrobials and supportive therapy are often adopted treatments for pyometra cases, surgery is not always selected in elderly dogs (Hagman, 2022). Antimicrobial resistance (Bellato et al., 2022; Lansubsakul et al., 2022; Lord et al., 2023), including multidrug resistance (MDR) involving three or more classes (Magiorakos et al., 2012), is a concerning reality. MDR pathogens have caused widespread infections in dogs (El‐Tarabili et al., 2022; Gibson et al., 2008; Silva et al., 2018), as observed in limited studies on pyometra (Agostinho et al., 2014). Therefore, these pathogens are of clinical and epidemiological relevance due to their potential for animal‐to‐animal transmission (Xavier, Santana, et al., 2022), particularly in settings involving close contact between infected animals and humans (Miranda et al., 2021).

The aim of this study was to characterise the pathogenicity and MDR profiles of bacteria isolated from bitches with pyometra and to assess the contribution of different anatomical sites to uterine contamination.

## MATERIALS AND METHODS

### Demographics and clinical signs

This was a prospective observational study, including 23 female dogs diagnosed with pyometra and undergoing OSH at the Veterinary Hospital of the Federal University of Jataí (UFJ) and private veterinary clinics in Jataí, Goiás (GO). This project was approved by the Ethics Committee on Animal Use of UFJ (CEUA/UFJ), under registration number 006/2018. All dog owners provided written informed consent for their dogs to participate in the study. Information on breed, age category (Young, Adult, Senior, Geriatric), reproductive history (Multiparous or Nulliparous), type of pyometra (Open, with purulent discharge from the vulva, or Closed, with no external discharge) and history of contraceptive drug use is presented in Table 1 to allow assessment of potential risk factors.

**Table 1:** Features of bitches with pyometra undergoing therapeutic ovariohysterectomy (OSH), including breed and age

Dog ID	Breed	Age		Parity (no.)	Pyometra	Contraceptive use (no.)
		Years	Category*			
1	Mongrel	8	Senior	Multiparous (2)	Open	No
2	Poodle	13	Geriatric	Multiparous (3)	Open	No
3	Poodle	7	Senior	Multiparous (1)	Open	No
4	Mongrel	4	Adult	Multiparous (2)	Closed	No
5	Pit Bull	4	Adult	Nulliparous	Open	No
6	Mongrel	5	Adult	Nulliparous	Open	Yes (1)
7	German shepherd	7	Senior	Multiparous (2)	Open	Yes (1)
8	Pinscher	5	Adult	Multiparous (1)	Open	No
9	Shih‐tzu	3	Adult	Nulliparous	Closed	No
10	Mongrel	11	Senior	Multiparous (2)	Closed	Yes (1)
11	Mongrel	7	Senior	Nulliparous	Open	Yes (1)
12	Poodle	6	Adult	Nulliparous	Open	No
13	Pit Bull	5	Adult	Nulliparous	Closed	Yes (1)
14	Mongrel	6	Adult	Nulliparous	Closed	Yes (1)
15	Mongrel	8	Senior	Nulliparous	Closed	Yes (1)
16	Poodle	13	Geriatric	Nulliparous	Open	No
17	Mongrel	10	Senior	Multiparous (1)	Closed	Yes (1)
18	Mongrel	5	Adult	Multiparous (1)	Closed	Yes (4)
19	Mongrel	2	Adult	Nulliparous	Closed	Yes (2)
20	Mongrel	2	Adult	Nulliparous	Closed	Yes (2)
21	Mongrel	5	Adult	Multiparous (2)	Closed	Yes (1)
22	Poodle	10	Senior	Multiparous (3)	Open	Yes (3)
23	Yorkshire terrier	6	Adult	Nulliparous	Closed	No

Parity: Multiparous/Nulliparous; Pyometra: Open/Closed

ID Identification, N.i. Not informed, no. Number of

* Age category based on the following criteria: young (<2 years old); adults (from 2 to 6 years old); seniors (from 7 to 11 years old) and geriatric (≥12 years old)

### Collection of biological samples

Biological samples were collected from all female dogs intraoperatively from four anatomical sites: uterine content was collected by needle puncture using a large‐calibre needle; rectal (faecal) and vaginal contents were collected using sterile individual swabs; and urine samples (3 mL) were collected by cystocentesis. The samples were inoculated into brain–heart infusion (BHI) liquid medium and incubated aerobically at 37°C for 24 hours.

### Isolation and microbiological classification

Biological samples were initially collected from all animals and incubated in BHI broth for 24 hours as a screening step. Only samples exhibiting bacterial growth after this incubation were considered culture‐positive and processed further. From each culture‐positive sample, aliquots were plated onto MacConkey agar using the streak plate technique and incubated aerobically at 37°C for 24 hours for the isolation of Gram‐negative bacteria. It is important to note that all subsequent analyses were performed at the isolate level rather than at the sample level. For each culture‐positive sample, up to three morphologically distinct colonies suggestive of *E. coli*, *K. pneumoniae*, *Proteus* spp. or *Enterobacter* spp. were selected following Gram staining and subjected to biochemical identification (Indole, Methyl Red, Voges–Proskauer, citrate, lysine, oxidase and glucose tests). Confirmed *E. coli* isolates were preserved on Conservation agar and stored in 50% glycerol for subsequent phylogenetic group determination and virulence gene analysis.

Culture aliquots from BHI broth were streaked onto Mannitol Salt agar plates and incubated aerobically at 37°C for 48 hours to isolate Gram‐positive bacteria. Three colonies of fermenting mannitol were selected for morphological analysis by Gram staining (cocci in clustered arrangement) and to verify their reaction in the presence of catalase. Based on these confirmations, biochemical tests (Coagulase and Voges–Proskauer) were performed to identify *Staphylococcus* spp. In addition to biochemical confirmation, only *E. coli* isolates were identified using molecular methods (PCR), as this bacterium was the primary focus of the study due to its high prevalence in canine pyometra.

### Antimicrobial susceptibility testing

Disc diffusion assays were performed on Mueller–Hinton agar using the following antibiotic groups: aminoglycosides (10 μg gentamicin, GEN); β‐lactam antibiotics (20 μg and 10 μg amoxicillin‐clavulanic acid, AMO + CLA, respectively; 10 μg imipenem, IPM; and 10 μg penicillin, PEN); cephalosporins (30 μg cefazolin, CFZ; cefalotin, 30 μg CFL; 30 μg ceftriaxone, CFX); fluoroquinolones (5 μg ciprofloxacin, CIP); macrolides (15 μg azithromycin, AZI); tetracycline (30 μg TET); and doxycycline (30 μg DOX). Penicillin (10 μg PEN) was tested only against Gram‐positive bacteria. The standard quality control (QC) strains used for antimicrobial susceptibility testing were *E. coli* (ATCC 25922), *K. pneumoniae* (ATCC 700603) and *S. aureus* (ATCC 29213), following CLSI guidelines. The diameter of the inhibition zone was measured after 24 hours of incubation at 37°C and classified as susceptible (S), intermediate (I) or resistant (R) according to Clinical and Laboratory Standards Institute (CLSI) guidelines (Xavier, Da Silva, et al., 2022) and as MDR, following Magiorakos et al. (2012).

### Phylogenetic classification of *E. coli* (Applied Biosystems, USA)

Bacterial DNA was obtained using the thermal extraction technique (Olsvick & Strockbine, 1993) and stored at −18°C for further analysis. Simultaneous DNA amplification (Quadruplex PCR) was performed to detect targets using primers *chuA, yjaA, TspE4.C2* and *arpA* (Table S1), following the methodology described by Clermont et al. (2013). The reactions were carried out in a final volume of 20 μL, composed of 2 μL of 10× PCR buffer [200 mM Tris–HCl (pH 8.4), 500 mM KCl], 3 mM MgCl_2_, 0.4 μL of 0.2 mM dNTP, 2 U of Taq polymerase, 3 μL of DNA sample and appropriate primers – 0.2 μM of each primer was used, except for primer *AceK.f* (0.4 μM). The Veriti 96‐Well thermocycler was used with initial denaturation at 94°C for 4 minutes, followed by 30 cycles at 94°C (5 seconds), 59°C (20 seconds) and 72°C (10 seconds). After completion of these cycles, a final extension was performed at 72°C for 5 minutes.

PCR‐amplified products were detected using 2% agarose gel electrophoresis (BioAmerica Biotech®) in 1× Tris/Borate/EDTA buffer (TBE: Tris 0.89 M; EDTA 0.02 M; boric acid 0.89 M), supplemented with a marker (GeneRuler® 1 kb DNA Ladder, Thermo Fisher, USA). After electrophoresis, gels were stained with 0.5 mg/mL ethidium bromide solution, visualised under a UV transilluminator and photographed using Gel Doc‐Print VX2. Amplified fragments with molecular weights (bp) of 400 bp (*arpA*), 288 bp (*chuA*), 211 bp (*yjaA*) and 152 bp (*TsE4.C2*) were considered positive for these genes.

### Virulence factor genes of *E. coli*

Isolates of *E. coli* from uterine contents were analysed for the presence of VFs using PCR targeting the papC, fimH, uspA and hlyA genes, with specific amplification primers (Table S2). The analysis was restricted to uterine isolates, as these represent the primary site of infection in pyometra and are most directly associated with disease pathogenesis and clinical outcome. The amplification reaction was carried out separately in a final volume of 30 μL, comprising 1× PCR buffer (200 mM Tris–HCl (pH 8.4), 500 mM KCl), 2 mM MgCl_2_, 0.2 mM of each dNTP, 1 U of Taq polymerase, 5 μL of DNA and 0.4 μM of each appropriate primer. A Veriti 96‐Well thermocycler was used with initial denaturation at 94°C for 10 minutes, followed by 30 cycles at 94°C (1 minute), annealing at 58°C (primers fimH and papC) and 63°C (primers hlyA and uspA) for 20 seconds and extension at 72°C for 2 minutes. After completion of these cycles, a final extension was performed at 72°C for 7 minutes.

PCR‐amplified products were detected using 2% agarose gel electrophoresis (Bioamerica Biotech®) in 1× Tris/Borate/EDTA buffer (TBE: 0.89 M Tris, 0.02 M EDTA, 0.89 M boric acid). After electrophoresis, gels were stained with 0.5 mg/mL ethidium bromide solution, visualised under UV transilluminator and recorded using Gel DOC‐Print VX2® gel documentation‐imaging system.

### Statistical analysis

All statistical analyses were conducted using Sigma Plot v. 11 software (Systat Software Inc., USA) using linear regression analysis. Results were considered significant when *P* < .05 (two‐tailed).

## RESULTS

### Risk factors for the prevalence of pyometra in female dogs

Female dogs over 6 years of age were less frequently observed with closed pyometra compared with younger animals, and dogs with a history of contraceptive use appeared more often to present with closed pyometra. No statistically significant association was found between the type of pyometra (open or closed) and bacterial pathogen or number of pregnancies (Table 2). These observations should be interpreted with caution, as the absence of a comparison population represents a limitation of the present study.

**Table 2:** Association of clinical variables in female dogs with pyometra, based on logistic regression*

Variables	Coefficient	*P*	Odds ratio	Confidence interval (95%)
Age^‡^	−1.5224	.05	0.21	0.04 to 1.06
Contraceptive use	1.2993			
Contraceptive use^§^	1.3499	.06	3.85	0.96 to 17.32
Intercept	−0.2513			
Bacterial pathogen^#^	−0.7157	.36	0.48	0.10 to 2.31
Pregnancy^¶^	−1.4021	.14	0.24	0.04 to 1.60
Intercepto	1.8339			

* Dependent variable: open (0) and closed (1) pyometra;

Independent variables: ^‡^Age: young (0) and adult (1); ^§^Contraceptive: no (0) and yes (1); ^#^Bacterial pathogen: absent (0) and present (1); ^¶^Pregnancy: nulliparous (0) and multiparous (1); no statistically significant differences was observed in other associations among parameters (breed, pyometra type, pregnancy status)

### *Escherichia coli* predominated in canine pyometra, followed by *Staphylococcus* spp.

**FIG 1:**
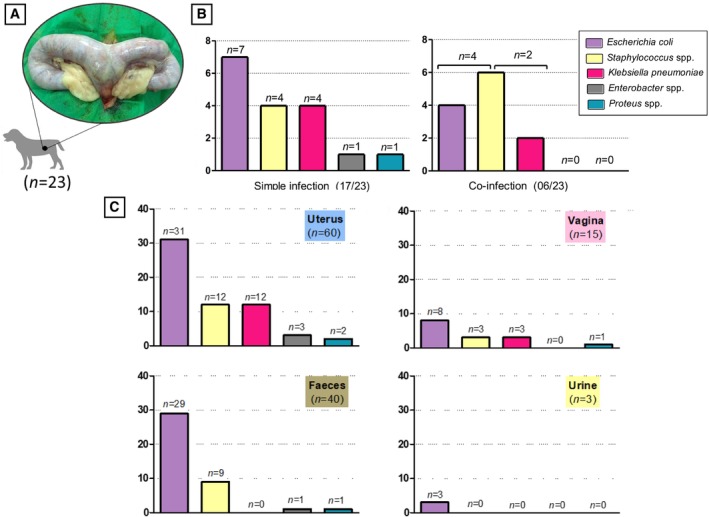
Female dogs with pyometra that underwent ovariohysterectomy (OSH, *n* = 23) had biological samples collected from uterine, vaginal, faecal and urinary contents, which were cultured for bacteriological identification based on conventional methods, such as macroscopic and microscopic morphology and biochemical tests (A). Single infections (17/23) and coinfections (6/23) with *Escherichia coli, Staphylococcus* spp., *Klebsiella pneumoniae, Enterobacter* spp. and *Proteus* spp. were identified among the dogs, with *E. coli* being the prevalent pathogen (B). Of the total isolates identified (*n* = 118) in different anatomical sites [uterus (*n* = 60), vaginal (*n* = 15), faecal (*n* = 40) and urine (*n* = 3)], *E. coli* was the prevalent pathogen (*n* = 71), followed by *Staphylococcus* spp. (*n* = 24), *K. pneumoniae* (*n* = 15), *Enterobacter* spp. (*n* = 4) and *Proteus* spp. (*n* = 4) (C).

After incubation in BHI broth, bacterial growth was detected in all uterine samples, whereas only culture‐positive vaginal, faecal and urinary samples were included in further analyses. The results showed that 17 out of 23 female dogs with pyometra (73.9%, Fig 1A) presented single infections, while six dogs (6/23, 26.1%) had uterine coinfections (Fig 1B). *Escherichia coli* (7/23, 30.4%) was the predominant pathogen, followed by *Staphylococcus* spp. (4/23, 17.4%), *K. pneumoniae* (4/23, 17.4%), *Enterobacter* spp. (1/23, 4.3%) and *Proteus* spp. (1/23, 4.3%). Conversely, coinfections involved *E. coli* + *Staphylococcus* spp. (4/23, 17.4%) and *Staphylococcus* spp. + *K. pneumoniae* (2/23, 8.7%).

### *Escherichia coli* predominated among samples from different anatomical sites

In total, 118 isolates were identified in the analysed samples: *E. coli* (71/118, 60.2%), *K. pneumoniae* (15/118, 12.7%), *Enterobacter* spp. (4/118, 3.4%), *Proteus* spp. (4/118, 3.4%) and *Staphylococcus* spp. (24/118, 20.3%). *Escherichia coli* predominated at all anatomical sites, comprising over half of uterine and vaginal isolates, over 70% of faecal isolates, and all urinary isolates. Other genera occurred at lower and variable frequencies (Fig 1C).

### Multidrug resistance among pathogens causing canine pyometra

**FIG 2:**
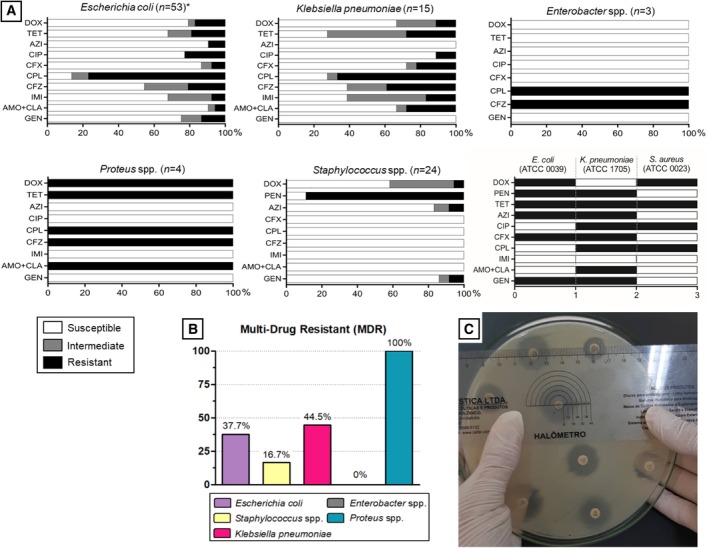
*In vitro* susceptibility (%) of *Escherichia coli, Klebsiella pneumoniae, Enterobacter* spp., *Proteus* spp. and *Staphylococcus* spp. isolated from uterine, vaginal, faecal and urinary contents of female dogs with pyometra, as well as standard strains (ATCC 0039, ATCC 1705 and ATCC 0023), to major veterinary antimicrobials: doxycycline (DOX); tetracycline (TET); azithromycin (AZI); ciprofloxacin (CIP); ceftriaxone (CFX); cephalothin (CPL); cefazolin (CFZ); imipenem (IMI); amoxicillin‐clavulanate (AMO + CLA); gentamicin (GEN); and penicillin (PEN). The pathogens were classified as susceptible (white), intermediate (grey) and resistant (black, A), according to CLSI (2019); and as multidrug‐resistant (coloured, B), according to Magiorakos et al. (2012), by reading their inhibition zones based on the disc diffusion technique (C). *53 out of *71 E. coli* isolates were tested due to resource limitations.

Based on antimicrobial susceptibility (Fig 2A), *E. coli* was largely susceptible to AMO + CLA, AZI and CFX, but highly resistant to CPL. In turn, *K. pneumoniae* showed full susceptibility to AZI and GEN, high susceptibility to CIP, and notable resistance to CPL and CFZ. *Enterobacter* spp. isolates were resistant only to CPL and CFZ, whereas *Proteus* spp. were susceptible to AZI, CIP, IMI and GEN but resistant to several other drugs. *Staphylococcus* spp. isolates were broadly susceptible, except for high resistance to PEN.

Regarding MDR (Fig 2B), 37.7% of *E. coli* and 44.5% of *K. pneumoniae* isolates were MDR, with some additionally resistant to a fourth antimicrobial class. No MDR was detected in *Enterobacter* spp., whereas all *Proteus* spp. isolates were MDR. Among *Staphylococcus* spp., 16.7% showed MDR. Inhibition zone measurements are shown in Fig 2C.

### Phylogenetic relatedness and virulence profile of *E. coli* isolates

**FIG 3:**
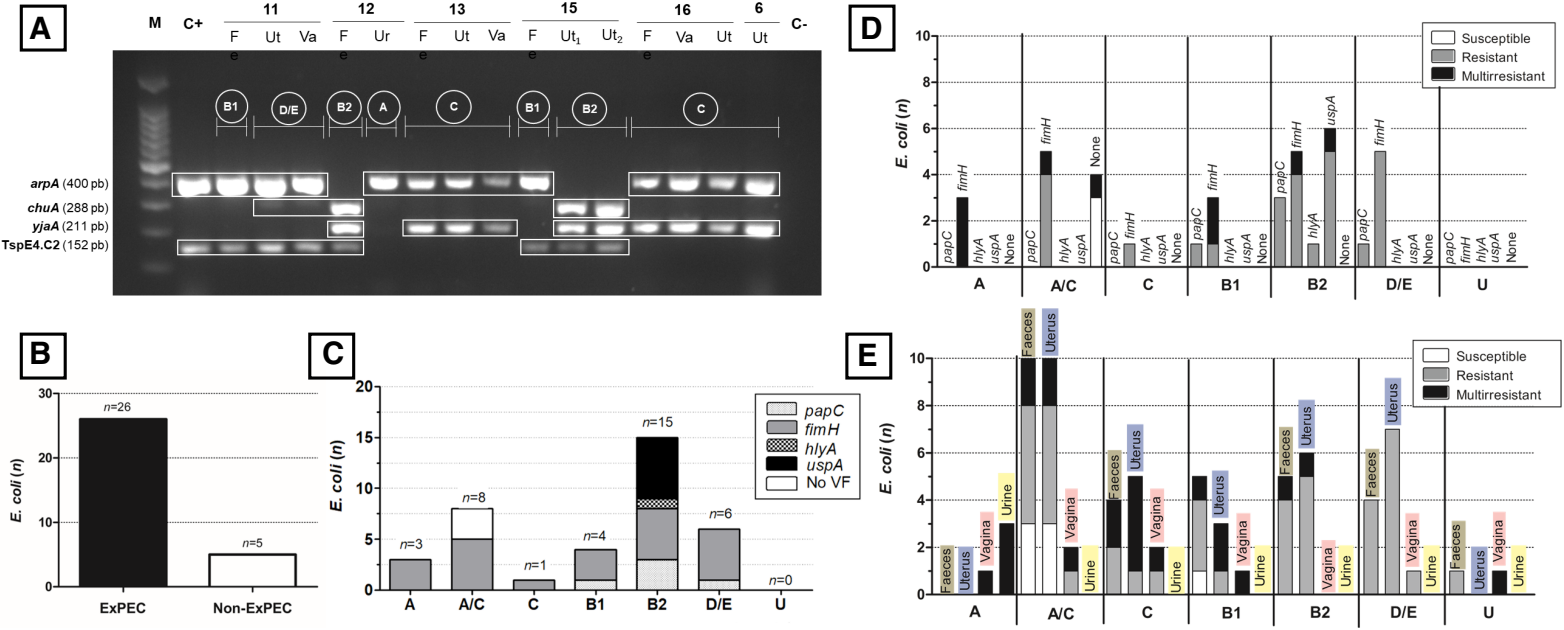
Phylogenetic analysis of 71 *Escherichia coli* isolates from uterine (Ut, *n* = 31), vaginal (Va, *n* = 8), faecal (F, *n* = 29) and urinary (Ur, *n* = 3) samples from bitches with pyometra. Quadruplex PCR using chuA, yjaA, TspE4.C2 and arpA primers was followed by 2% agarose gel electrophoresis, with ethidium bromide confirming amplified fragments of 400 bp (arpA), 288 bp (chuA), 211 bp (yjaA) and 152 bp (TspE4.C2) in animals (canine ID) 6, 11, 12, 13, 1 16 (A). Uterine isolates were classified as ExPEC or non‐ExPEC (B) and screened for virulence genes papC, fimH, hlyA and uspA (C). Antimicrobial susceptibility of virulence factor (VF)‐positive isolates (D) and the anatomical distribution of resistant and multidrug‐resistant isolates (E) are shown.

Given the predominance of *E. coli*, a phylogenetic analysis of 71 isolates from uterine, vaginal, faecal and urinary samples was performed, with all isolates amplifying at least one gene (Fig 3A). In 90% (10/11) of dogs, the same *E. coli* phylogroup was detected in at least two anatomical sites (Table S3), indicating a likely clonal relationship within each animal. Multiple phylogroups were occasionally found across reproductive and rectal sites. Urinary isolates and phylogroup U showed no clonal link with other sites. These findings suggest the intestine as a major reservoir of *E. coli* and support a possible ascending route from the vagina to the uterus in pyometra. Most uterine *E. coli* isolates were ExPEC (26/31; 83.9%, Fig 3B). These isolates carried at least one VF (83.9%, 26/31), predominantly fimH. Strains with multiple VF genes were mainly from phylogroup B2, with only one isolate harboring all four genes and one no‐VF strain detected. All phylogroups except U showed at least one VF, and B2 uniquely included strains positive for all VFs (Fig 3C).

### Antimicrobial resistance and multidrug resistance in *E. coli* isolates

Drug resistance occurred in 77.4% (24/31) of uterine *E. coli* isolates, with eight classified as MDR, mainly from phylogroup B2 and VF‐positive. Five dogs were infected with MDR strains (Table S4). All VF‐positive isolates showed resistance or MDR, and all phylogroups except U were at least fimH‐positive and resistant (Fig 3D). One no‐VF isolate was MDR, suggesting additional resistance mechanisms. Regarding each anatomical site, MDR *E. coli* were detected across all phylogroups except D/E (24/71), predominantly in uterine samples, followed by faecal, vaginal and urinary sites (Fig 3E).

## DISCUSSION

Canine pyometra remains a frequent and potentially life‐threatening condition in small animal practice, requiring rapid clinical decision‐making regarding antimicrobial therapy and surgical intervention. In this context, understanding the aetiological agents involved, their antimicrobial susceptibility profiles and their potential reservoirs is essential to optimise empirical treatment, improve perioperative management and mitigate the risks associated with antimicrobial resistance. The present study provides clinically relevant data on the bacterial composition of pyometra across multiple anatomical sites and highlights resistance patterns with direct implications for therapeutic strategies in veterinary medicine.

Although contraceptive use appeared clinically relevant among the evaluated variables, the available data did not support a meaningful analytical assessment of this factor within the scope of the present study. Consistent with previous studies, *E. coli* was the main aetiological agent of canine pyometra, occurring in both single and mixed infections across all anatomical sites (Hagman, 2022). *Staphylococcus* spp. and *Klebsiella* spp. were also frequently isolated, whereas *Enterobacter* spp. and *Proteus* spp. were less common, as reported elsewhere (Huber et al., 2022; Lansubsakul et al., 2022; Zheng et al., 2023). Although coinfections were less prevalent, their occurrence highlights increased microbiological complexity and potential therapeutic challenges.

Several antimicrobials commonly used in veterinary practice remain effective against pathogens associated with pyometra; however, resistance to cephalosporins, beta‐lactams and tetracyclines was frequent, particularly among Gram‐negative bacteria. These findings are consistent with reports from both veterinary and human medicine, emphasising concerns regarding the declining efficacy of previously reliable drugs (Bandyopadhyay & Mukherjee, 2022; Bellato et al., 2022).

Despite the importance of culture and susceptibility testing (Hagman, 2022), the acute and potentially life‐threatening nature of pyometra often necessitates empirical antimicrobial therapy (Lavin & Maki, 2023). In this context, the detection of MDR *E. coli*, *Staphylococcus* spp., *K. pneumoniae* and *Proteus* spp. underscores the need for prompt diagnostic testing to guide therapeutic decisions. Although surgery remains the treatment of choice (Hagman, 2022; Liao et al., 2020), recent evidence highlights that the benefit of postoperative antimicrobial therapy in uncomplicated pyometra is not yet established (Ylhäinen et al., 2023), emphasising the importance of targeted therapy guided by susceptibility testing and One Health considerations.

Multidrug resistance to three or more antimicrobial classes (Magiorakos et al., 2012) was observed in all bacterial genera isolated from female dogs in this study, except *Enterobacter* spp., with rates ranging from 16.7% to 100% (Fig 2A), consistent with previous reports for *E. coli* (Agostinho et al., 2014), *Enterobacter* spp. (Gibson et al., 2008), *K. pneumoniae* (Silva et al., 2018), *Proteus* spp. (El‐Tarabili et al., 2022) and *Staphylococcus* spp. (Lord et al., 2023). *Escherichia coli* predominated across anatomical sites, with phylogroup A/C being the most frequent, although B2 – associated with pyometra and animal transmission (Mateus et al., 2013; Xavier, Santana, et al., 2022) – was detected in 15.5% of samples (11/71) from four bitches. Faecal and reproductive isolates often shared clonal lineages, highlighting the intestinal microbiota as a reservoir facilitating uterine colonisation. Virulence genes fimH, papC, hlyA and uspA were detected, particularly in B2 isolates, with fimH widespread across phylogroups and crucial for adherence to the endometrium (Krekeler et al., 2012). HlyA and uspA contributed to cytotoxicity and stress resilience (Bandyopadhyay & Mukherjee, 2022; Johnsen et al., 2019; Menestrina et al., 1994). Alarmingly, seven of the 11 *E. coli*–infected dogs harboured MDR strains, mainly phylogroup C carrying fimH, underscoring that female dogs with pyometra can serve as reservoirs for MDR *E. coli* in reproductive, excretory and other potentially MDR genera, complicating clinical management in veterinary practice.

In humans, antimicrobial resistance poses a significant challenge to public health, particularly among susceptible populations such as the elderly, who are more vulnerable to adverse outcomes and comorbidities (GBD, 2024). This issue has global relevance, as resistant microorganisms can compromise medical procedures, including childbirth, surgery and chemotherapy in different healthcare settings worldwide (Davies, 2018; Sprenger, 2017). Within this broader context, the present study demonstrates that bitches with pyometra may harbour MDR bacteria across different anatomical sites, including the intestinal microbiota.

In conclusion, *E. coli* was the predominant bacterial species associated with canine pyometra, occurring in both single and polymicrobial infections, followed by *Staphylococcus* spp., *K. pneumoniae*, *Enterobacter* spp. and *Proteus* spp. The frequent identification of identical *E. coli* phylogroups in faecal and reproductive samples supports the intestinal microbiota as a relevant reservoir and a likely source of ascending uterine infection. Most uterine *E. coli* isolates carried at least one virulence‐associated gene, predominantly *fimH*, consistent with an extraintestinal pathogenic profile. Antimicrobial resistance was common, with MDR detected in isolates from all genera except *Enterobacter* spp. Taken together, these findings highlight the microbiological complexity of pyometra and reinforce the importance of culture and antimicrobial susceptibility testing to support rational antimicrobial use and optimise clinical management in female dogs with this condition.

### Author contributions

**M. G. M. Camozzi:** Conceptualisation, methodology, investigation, formal analysis, writing – original draft preparation, reviewing and editing. **S. B. Waller:** Formal analysis, investigation, writing – original draft preparation; writing – review and editing. **E. C. Castelhano:** Methodology, investigation. **A. L. S. Neves:** Methodology. **B. R. Arrais:** Resources, formal analysis. **J. F. N. Pinto:** Resources, formal analysis. **B. G. Alves:** Formal analysis, investigation. **C. N. Moreira:** Conceptualisation, investigation, formal analysis, writing – review and editing, supervision. All authors approved the final manuscript.

### Conflict of interest

The authors declare that they have no known competing financial interests or personal relationships that could have appeared to influence the work reported in this paper.

## Supporting information


**Table S1.** Primers used in PCR for phylogenetic classification of *Escherichia coli* strains isolated from bitches with pyometra
**Table S2**. Primers used to amplify virulence factors in *Escherichia coli* isolated from bitches with pyometra
**Table S3**. Phylogenetic groups of *Escherichia coli* identified through quadruplex PCR applied to 71 isolates of uterine (*n* = 31), faecal (*n* = 29), vaginal (*n* = 8) and urinary (*n* = 3) samples derived from 11 bitches with pyometra
**Table S4**. Genes encoding virulence factors *hlyA*, *uspA*, *fimH* and *papC* in *Escherichia coli* isolated from the uteri (*n* = 31) of bitches with pyometra and their association with the incidence of extraintestinal pathogenic *E. coli* (ExPEC), as well as their genetic phylogroups and antimicrobial susceptibility

## Data Availability

The participants of this study did not give written consent for their data to be shared publicly, so supporting data are not available.
