# Supplementary material for: Multidrug resistance of bacterial pathogens in canine pyometra
Source: J Small Anim Pract. 2026 Mar 16;67(7):627–34. doi: 10.1111/jsap.70110 (PMC13327223; doi:10.1111/jsap.70110)
Supplement: Supplementary file 1 — Table S1. Primers used in PCR for phylogenetic classification of Escherichia coli strains isolated from bitches with pyometra Table S2. Primers used to amplify virulence factors in Escherichia coli isolated from bitches with pyometra Table S3. Phylogenetic groups of Escherichia coli identified through quadruplex PCR applied to 71 isolates of uterine (n = 31), faecal (n = 29), vaginal (n = 8) and urinary (n = 3) samples derived from 11 bitches with pyometra Table S4. Genes encoding virulence factors hlyA, uspA, fimH and papC in Escherichia coli isolated from the uteri (n = 31) of bitches with pyometra and their association with the incidence of extraintestinal pathogenic E. coli (ExPEC), as well as their genetic phylogroups and antimicrobial susceptibility [file JSAP-67-627-s001.pdf]

Supplementary Table 1. Primers used in PCR for phylogenetic classification of *Escherichia coli* strains isolated from bitches with pyometra.

Gene	Primer ID	Primer sequence	Amplification (bp)
chuA	chuA.1b	ATGGTACCGGACGAACCAAC	288
	chuA.2	TGCCGCCAGTACCAAAGACA	
yjaA	yjaA.1b	CAAACGTGAAGTGTCAGGAG	211
	yjaA.2b	AATGCGTTCCTCAACCTGTG	
TspE4.C2	TspE4.C2.1b	CACTATTCGTAAGGTCATCC	152
	TspE4.C2.2b	AGTTTATCGCTGCGGGTCGC	
arpA	AceK.f	AACGCTATTCGCCAGCTTGC	400
	ArpA1.r	TCTCCCCATACCGTACGCTA	

ID, identification; bp, base pairs; methodology based on Clermont et al. (2013).

Supplementary Table 2. Primers used to amplify virulence factors in *Escherichia coli* isolated from bitches with pyometra.

Virulence factor			Amplification (bp)	Reference
Gene ID	Direction	Primer sequence (5'–3')		
papC	Frw	GACGGCTGTACTGCAGGGTGTGGG	328	Le Bouguenec et al. (1992)
	Rev	ATATCCTTTCTGCAGGGATGCAATA		
fimH	Frw	TGCAGAACGGATAAGCCGTGG	508	Johnson and Stell (2000)
	Rev	GCAGTCACCTGCCCTCCGGTA		
uspA	Frw	ATGCTACTGTTTCCGGGTAGTGTGT	1000	Nakano et al. (2001)
	Rev	CATCATGTAGTCGGGGCGTAACAAT		
hlyA	Frw	AACAAGGATAAGCACTGTTCTGGCT	1177	Yamamoto et al. (1995)
	Rev	ACCATATAAGCGGTCATTCCCGTCA		

ID, identification; bp, base pairs; Frw, Forward; Rev, Reverse.

Supplementary Table 3. Phylogenetic groups of *Escherichia coli* identified through quadruplex PCR applied to 71 isolates of uterine (*n*=31), faecal (*n*=29), vaginal (*n*=8) and urinary (*n*=3) samples derived from 11 bitches with pyometra.

**Canine ID***	**Samples**	**Phylogenetic groups**							**Overall**
		**A**	**A/C**	**C**	**D/E**	**B1**	**B2**	**U**	
**1**	Faecal		2			1			**3**
	Uterine								**-**
	Vaginal								**-**
	Urinary								**-**
**3**	Faecal				3				**3**
	Uterine				5				**5**
	Vaginal								**-**
	Urinary								**-**
**5**	Faecal						3	1	**4**
	Uterine						3		**3**
	Vaginal				1				**1**
	Urinary								**-**
**6**	Faecal				1	2			**3**
	Uterine		1	1					**2**
	Vaginal		1					1	**2**
	Urinary								**-**
**7**	Faecal		3	1					**4**
	Uterine		3	1					**4**
	Vaginal								**-**
	Urinary								**-**
**8**	Faecal		2	1					**3**
	Uterine	1	2						**3**
	Vaginal								**-**
	Urinary								**-**
**11**	Faecal		2			1			**3**
	Uterine		1		2				**3**
	Vaginal	1				1			**2**
	Urinary								**-**
**12**	Faecal		1				1		**2**
	Uterine		3						**3**
	Vaginal								**-**
	Urinary	3							**3**
**13**	Faecal	1							**1**
	Uterine	1				1			**2**
	Vaginal		1	1					**2**
	Urinary								**-**
**15**	Faecal					1			**1**
	Uterine					1	1		**3**
	Vaginal								**-**
	Urinary								**-**
**16**	Faecal	1					1		**2**
	Uterine	1				1	1		**3**
	Vaginal			1					**1**
	Urinary								-
**Overall (n=11)**	***n***	**9**	**22**	**6**	**12**	**9**	**11**	**2**	**71**
	**%**	**12.7%**	**30.9%**	**8.5%**	**16.9%**	**12.7%**	**15.5%**	**2.8%**	**100%**

* *E. coli* was isolated from 11 of the 23 female dogs with pyometra; these isolates were included in the phylogenetic study, according to Clermont et al. (2013); ID, identification; U, unknown.

Supplementary Table 4. Genes encoding virulence factors *hlyA*, *uspA*, *fimH* and *papC* in *Escherichia coli* isolated from the uteri (n=31) of bitches with pyometra and their association with the incidence of extraintestinal pathogenic *Escherichia coli* (ExPEC), as well as their genetic phylogroups and antimicrobial susceptibility.

**Canine ID***	**Uterine sample ID**	**Genes encoding virulence factors**					**ExPEC**	**Phylogroup**	**Antimicrobial Susceptibility**
		hlyA	***uspA***	***fimH***	***papC***	**Overall genes**			
	3a			+		1	Yes	D/E	R
	3b			+	+	2	Yes	D/E	R
**3**	3c			+		1	Yes	D/E	R
	3d			+		1	Yes	D/E	×
	3e			+		1	Yes	D/E	×
	5a		+	+	+	3	Yes	B2	R
**5**	5b		+	+		2	Yes	B2	R
	5c		+	+	+	3	Yes	B2	R
**6**	6a			+		1	Yes	C	R
	6b			+		1	Yes	A/C	MDR
	7a			+		1	Yes	C	×
**7**	7b			+		1	Yes	A/C	R
	7c			+		1	Yes	A/C	R
	7d			+		1	Yes	A/C	R
	8a			+		1	Yes	A	MDR
**8**	8b			+		1	Yes	A/C	R
	8c					0	-	A/C	MDR
	11a			+		1	Yes	D/E	R
**11**	11b			+		1	Yes	D/E	R
	11c					0	-	A/C	×
	12a					0	-	A/C	S
**12**	12b					0	-	A/C	S
	12c					0	-	A/C	S
**13**	13a			+	+	2	Yes	B1	R
	13b			+		1	Yes	A	MDR
	15a	+	+	+	+	4	Yes	B2	R
**15**	15b		+	+	+	3	Yes	B2	R
	15c			+		1	Yes	B1	MDR
	16a			+		1	Yes	A	MDR
**16**	16b			+		2	Yes	B1	MDR
	16c		+	+		1	Yes	B2	MDR
Overall bitches (n=10)	Overall sample (n=31)	1	6	28	6	0 gene: 5<br>1 gene: 18<br>2 genes: 4<br>3 genes: 3<br>4 genes: 1	Yes: 26<br>No: 5	A: 3<br>A/C: 10<br>C: 2<br>B1: 3<br>B2: 6<br>D/E: 7<br>U: 0	S: 3<br>R: 16<br>MDR: 8<br>×: 4

* *E. coli* was isolated from 11 out of 23 female dogs with pyometra; samples collected from 10 of them were included in the study focusing on virulence factor genes (g.); ID, identification; *hlyA*, α-haemolysin; *uspA*, universal stress protein A; *fimH*, mannose-specific adhesin on type 1 fimbriae; *papC*, P fimbriae-encoding gene; +, presence of encoding gene; empty cell, absence of encoding gene; "-", non-ExPEC; phylogenetic groups were classified based on Clermont et al. (2013); antimicrobial susceptibility was classified as susceptible (S) and resistant (R), based on CLSI (2019), and as multidrug-resistant (MDR), based on Magiorakos et al. (2012); ×, no antimicrobial assay was performed.

References

Bouguenec, C. L., Archambaud, M. & Labigne, A. (1992) Rapid and specific detection of the pap, afa, and sfa adhesin-encoding operons in uropathogenic *Escherichia coli* strains by polymerase chain reaction. *Journal of Clinical Microbiology,* **30**, 1189–1193. https://doi.org/10.1128/jcm.30.5.1189-1193.1992

Clermont, O., Christenson, J.K., Denamur, E. & Gordon, D.M. (2013) The Clermont *Escherichia coli* phylo-typing method revisited: improvement of specificity and detection of new phylo-groups. *Environmental Microbiology Reports,* **5**, 58–65. https://doi.org/10.1111/1758-2229.12019

CLSI, Clinical and Laboratory Standards Institute (2019) Performance Standards for Antimicrobial Susceptibility Testing, 29th ed., CLSI supplement M100, Wayne, Pennsylvania, USA.

Johnson, J.R. & Stell, A.L. (2000) Extended virulence genotypes of *Escherichia coli* strains from patients with urosepsis in relation to phylogeny and host compromise. *Journal of Infectious Diseases,* **181**, 261-272. https://doi.org/10.1086/315217

Magiorakos, A.P., Srinivasan, A., Carey, R.B., Carmeli, Y., Falagas, M.E., Giske, C.G., Harbarth, S., Hindler, J.F., Kahlmeter, G., Olsson-Liljequist, B., Paterson, D.L., Rice, L.B., Stelling, J., Struelens, M.J., Vatopoulos, A., Weber, J.T. & Monnet, D.L. (2012) Multidrug-resistant, extensively drug-resistant and pandrug-resistant bacteria: an international expert proposal for interim standard definitions for acquired resistance. *Clinical Microbiology and Infection,* **18**, 268–281. https://doi.org/10.1111/j.1469-0691.2011.03570.x

Nakano, M., Yamamoto, S., Terai, A., Ogawa, O., Makino, S., Hayashi, H., Nair, G.B. & Kurazono, H. (2001) Structural and sequence diversity of the pathogenicity island of uropathogenic *Escherichia coli* with encodes de USP protein. *FEMS Microbiology Letters,* **205**, 71–76. https://doi.org/10.1111/j.1574-6968.2001.tb10927.x

Yamamoto, S., Terai, A., Yuri, K., Kurazono, H., Takeda, Y. & Yoshida, O. (1995) Detection of urovirulence factors in *Escherichia coli* by multiplex polymerase chain reaction. *FEMS Immunology and Medical Microbiology,* **12**, 85–90. https://doi.org/10.1111/j.1574-695X.1995.tb00179.x
